# Checkered Films of Multiaxis Oriented Nanocelluloses by Liquid-Phase Three-Dimensional Patterning

**DOI:** 10.3390/nano10050958

**Published:** 2020-05-18

**Authors:** Kojiro Uetani, Hirotaka Koga, Masaya Nogi

**Affiliations:** The Institute of Scientific and Industrial Research, Osaka University, Mihogaoka 8-1, Ibaraki-shi, Osaka 567-0047, Japan; hkoga@eco.sanken.osaka-u.ac.jp (H.K.); nogi@eco.sanken.osaka-u.ac.jp (M.N.)

**Keywords:** nanofiber alignment, birefringence, retardation, thermal transport

## Abstract

It is essential to build multiaxis oriented nanocellulose films in the plane for developing thermal or optical management films. However, using conventional orientation techniques, it is difficult to align nanocelluloses in multiple directions within the plane of single films rather than in the thickness direction like the chiral nematic structure. In this study, we developed the liquid-phase three-dimensional (3D) patterning technique by combining wet spinning and 3D printing. Using this technique, we produced a checkered film with multiaxis oriented nanocelluloses. This film showed similar retardation levels, but with orthogonal molecular axis orientations in each checkered domain as programmed. The thermal transport was enhanced in the domain with the oriented pattern parallel to the heat flow. This liquid-phase 3D patterning technique could pave the way for bottom-up design of differently aligned nanocellulose films to develop sophisticated optical and thermal materials.

## 1. Introduction

Nanocelluloses extracted from natural resources have large dimensional and functional anisotropy derived from their intrinsic extended chain crystal of cellulose I, so the various oriented structures of nanocelluloses have stunning properties in various materials including actuating plant cell walls [1], photonic structures with the chiral nematic phase [2,3], and fibers [4,5,6,7] or films [8,9] with high mechanical strength. In particular, recent studies have revealed that unidirectionally oriented nanocellulose films anisotropically conduct heat [10] or control optical retardation [11]. With regard to the thermal conductivity and optical controllability of nanocelluloses, for future paper electronics, the development of sophisticated materials such as phonon management film elements or polarizing arrays is expected by constructing fine multidirectional alignment structures within the film planes, rather than in the thickness direction like the self-assembling chiral nematic structure. For these purposes, it remains a great challenge to develop a practical orientation technique to multiaxis arrange the various nanocellulose alignments within the single film plane.

Various orientation techniques for nanocelluloses have been reported including shearing under evaporation [12,13], magnetic field orientation [14], biodirected epitaxial nanodeposition [15], wet spinning [4,5,6,7], cold drawing [8], doctor blade coating [9], gel stretching [10,11], evaporation-induced droplet casting [16,17], interfacial polyelectrolyte complex spinning [18], confinement-induced ordering [19], hydrodynamic alignment [20], and three-dimensional (3D) printing [21]. However, none of these methods can produce films with in-plane multiaxis oriented nanocelluloses because methods based on spinning, stretching, evaporation, and magnetic treatment only make the nanocellulose unidirectionally align. We need to combine the unidirectionally aligned nanocellulose parts to build multiaxis oriented nanocellulose films, but this is unrealistic because once dried, cellulose papers do not adhere to each other.

Only 3D printing is believed to be useful for preparing freely designed multiaxis oriented nanocellulose materials. However, 3D printing techniques often require the use of mixed inks with nanocelluloses and polymer matrices [22], so 100% nanocellulose material is difficult to obtain. When using a pure aqueous suspension of short cellulose nanocrystals (CNCs) from wood pulp, a very high concentration (20 wt%) was required to align the CNCs by shear force and to also have sufficient viscosity to make the patterns self-stand in air, and only thin wall structures were created in which the patterns were stacked in the *z*-axis direction [21]. As 2D film formation does not allow the gel width to shrink upon drying to enhance their orientations like the thin walls of 3D cellular architectures [21], it is believed to be very difficult to obtain two-dimensional (2D) films with in-plane multiaxis nanocellulose alignment.

To overcome the above problems, in this study, we developed the “liquid-phase” 3D patterning technique by combining wet spinning and 3D printing, which keeps unidirectionally aligned nanocellulose under wet condition to construct the multiaxis patterns. The nanocellulose suspensions were directly discharged to an acetone coagulation bath to form gels [23] to retain the oriented structures, and the never-dried patterned gels could adhere to each other by drying to form a single film. In addition, we used tunicate cellulose nanowhiskers (TNWs), known to have a higher aspect ratio than CNCs from wood pulp, in then expectation that they would align at lower concentrations with the application of lower pressure. By programming the orientation patterns, we succeeded in forming checkered films of multiaxis oriented nanocelluloses for the first time.

## 2. Materials and Methods

Two types of nanocelluloses were prepared from the tunicate of ascidian (*Halocynthia roretzi)* with reference to a previous study [24]. In brief, the tunicate of ascidian was cut into ~1 cm blocks and purified in a 1% NaClO_2_ aqueous solution with the addition of acetic acid at 80–90 °C for 5–6 h until the product became white. After washing, 3 g of the freeze-dried product was hydrolyzed in ~100 mL of 64% H_2_SO_4_ aqueous solution for 40 min. The suspension was washed with a large amount of distilled water to reach pH ~4. The suspension was then ultrasonicated for 30 min, and the supernatant was centrifuged at 3180 g for 60 min to obtain the TNWs. The sediments were agitated by a high-speed blender [25] at 37,000 rpm to fibrillate the tunicate cellulose nanofibers (TNFs). A drop of each of the diluted nanocellulose suspensions was dried on a cover glass and observed by scanning probe microscopy (SPM, SPM-9700HT, Shimadzu Corp., Kyoto, Japan) to image the nanocellulose appearance.

A gantry-type three-axis robot (BS-101005-1674, COMS Co. Ltd., Hyogo, Japan) with high positioning accuracy (± 2 µm) was created by custom order. A syringe pump (PHD ULTRA 70-3005, Harvard Apparatus, Holliston, MA, USA, or YSP-201, YMC Co. Ltd., Kyoto, Japan) was connected to the lock base needles (25G, 28G, and 30G with respective internal diameters of 0.25, 0.17, and 0.12 mm) with a 90° tip. A hydrophilic poly (tetrafluoroethylene) membrane filter (H010A090C or H010A142C, Advantec Toyo Kaisha, Ltd., Tokyo, Japan) was used for patterning the base materials to easily remove the nanocellulose gels or dried films from the glass Petri dish. The 2D x-ray diffraction (2D-XRD) measurements were outsourced to EAG Inc. (San Diego, CA, USA) in the same manner as previous studies [10,11]. A Siemens/Bruker GADDS system with a Hi-Star detector and a Huber goniometer was used with Cu Kα radiation (*λ* = 1.54059 Å) at 50 kV and 40 mA to obtain 2D diffraction frames five times at 200 s per frame, which were summed and averaged for better statistics without detector saturation issues. According to previous reports [4,9,12,18,21,26,27], the orientational order parameter *S* was calculated from the fitted intensity of the intensity distribution for the (200) plane of cellulose I crystals at 2*θ* = 22–23° with respect to the azimuthal angle *φ* using the following relations:

(1) $$S=\frac{3\langle \cos^{2}\phi_{c,z}\rangle -1}{2}$$

(2) $$\langle \cos^{2}\phi_{c,z}\rangle =1-2\langle \cos^{2}\phi_{200,z}\rangle$$

(3) $$\langle \cos^{2}\phi_{200,z}\rangle =\frac{\sum_{p}^{q}I\left(\phi \right)\sin\phi \cos^{2}\phi }{\sum_{p}^{q}I\left(\phi \right)\sin\phi }$$

The 2D retardation mapping images at the 543 nm wavelength were taken with a microscopy-type birefringence measurement system (WPA-micro, Photonic Lattice Inc., Miyagi, Japan) using a 2× objective lens. The average retardation and standard deviation were calculated from the retardation mapping data of 384 × 288 pixels in each image. The distribution of the slow axis corresponding to the higher refractive index direction (i.e., the cellulose chain direction) [11] was also extracted from each pixel data. Infrared thermography (CPA-E40, FLIR Systems Inc., Wilsonville, OR, USA) with preinstalled emissivity of 0.7 for paper was used to visualize the temperature distributions of the checkered films. The temperature profiles were extracted using QuickReport 1.2 SP2 software (FLIR Systems Inc., Wilsonville, OR, USA).

## 3. Results

### 3.1. Concept of Liquid-Phase 3D Patterning

**Figure 1:**
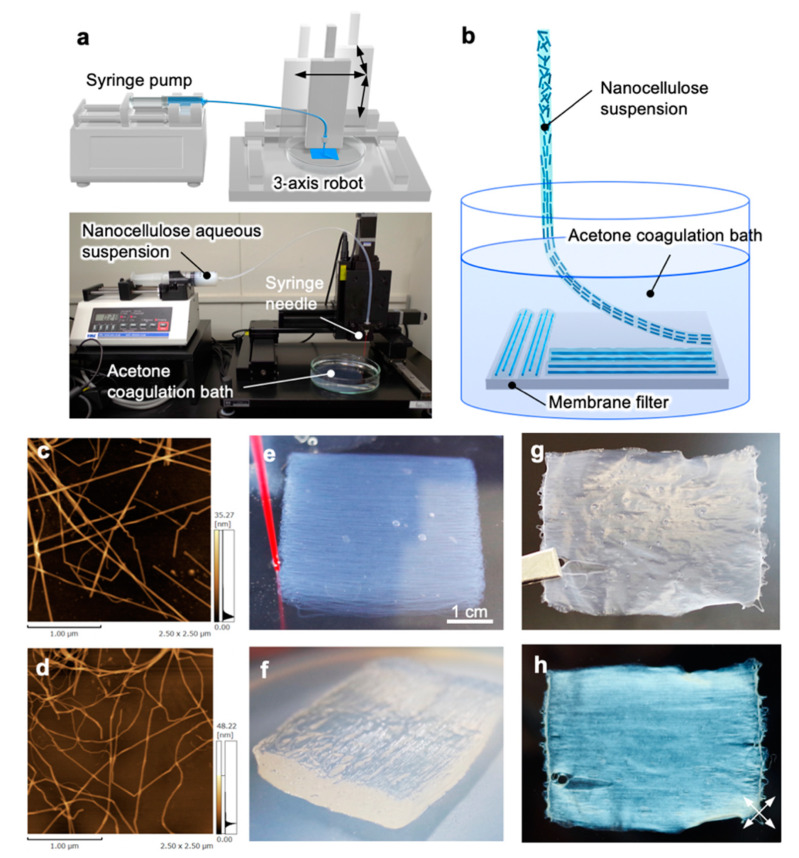
Liquid-phase 3D patterning of nanocelluloses. (**a**) Setup of the patterning system combining discharging of the nanocellulose suspension and patterning by the three-axis robot; (**b**) Conceptual illustration of patterning. SPM images of the (**c**) TNWs and (**d**) TNFs; (**e**) Nanocellulose gel during patterning in acetone; (**f**) Patterned nanocellulose gel removed from acetone; (**g**) Photograph and (**h**) crossed-Nicol view of the nanocellulose film after drying the patterned gel in (**f**) in an oven. The white double-headed arrows indicate the polarization axes.

We constructed the liquid-phase 3D patterning system by combining the discharging system of the nanocellulose aqueous suspension with the custom-made three-axis robot, as shown in Figure 1a. The nanocellulose suspension was directly discharged into the acetone coagulation bath to promote gelation to fix the orientation structures. In the case of normal 3D printing, the ejected material is brought into close contact with the support member or lower molded body to forcibly construct a 3D shape [22]. However, the liquid-phase patterning used in this study needed to have a certain distance between the needle and the bottom of the bath to maintain the gel shape and orientation of the nanocellulose, and the discharged gels were gently placed (Figure 1b). This system allows free design of nanocellulose gel patterns to build in-plane multiaxis oriented films.

We used two types of tunicate-derived nanocellulose: TNWs with a long straight morphology (Figure 1c) and TNFs with curved shapes (Figure 1d). For both nanocelluloses, the suspension with relatively low concentrations of 0.5–1.2 wt% was prepared for patterning. By setting the optimal discharging and patterning speeds, we succeeded in patterning the thin and fragile gels to programmed structures and then laminated them to the 3D gel aggregation (Figure 1e). After finishing patterning, the patterned gels had sufficient strength to pull them up from the acetone bath by a membrane filter laid in advance (Figure 1f) or by gently removing acetone with an electric pipettor. The raised gel on the membrane filter was dried in an oven to form a single free-standing film (Figure 1g). This film exhibited clear birefringence under crossed Nicols, showing the nanocellulose orientation in the patterned direction (Figure 1h).

### 3.2. Cooperation between Discharging and Patterning

As the nanocellulose discharging pump and three-axis robot are not digitally interconnected, it is important to determine the optimal discharging speed and patterning speed (head speed of the syringe) to achieve good patterning. We set various discharging and patterning speeds to judge the shape followability of the gel to the programmed pattern.

**Figure 2:**
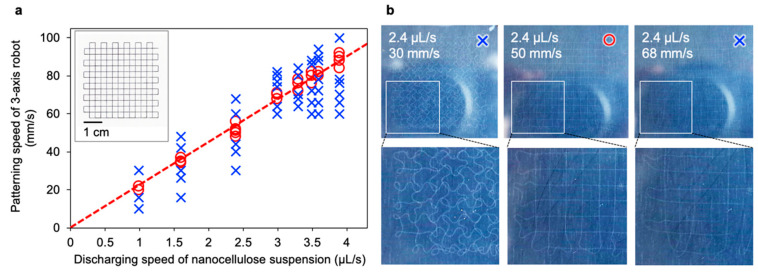
Optimal discharging and patterning speeds. (**a**) Mapping diagram of the patterning compatibility between the discharging speed of the nanocellulose suspension and the patterning speed using syringe needle 25G (internal diameter of 0.25 mm). The red circle and blue cross indicate the patterning results of the nanocellulose suspensions showing high and low followability to the programmed model pattern (insert), respectively. The dashed red line is the best fit to the red circles. The insert shows the one-stroke grid model for judging the drawing followability, which was drawn by the three-axis robot with a ballpoint pen on paper; (**b**) Patterned nanocellulose gels in acetone coagulation baths produced with various discharging and patterning speeds.

We defined the single-stroke lattice model pattern (insert of Figure 2a) and then evaluated the shape of each drawn gel. We found that sweet spots existed between the discharging and patterning speeds (red circles in Figure 2a). When the discharging speed was faster than the patterning speed, surplus gel was placed in a curved shape and did not have the programmed shape (left panel in Figure 2b). Conversely, when the patterning speed was too fast, the gel was dragged by the needles and broke in the middle (right panel in Figure 2b). The most balanced speeds allowed patterning as programmed (middle panel in Figure 2b). This tendency was observed regardless of the needle diameter or nanocellulose type. The accuracy of the folded portion of the gel was higher at a lower patterning rate. However, when the discharging speed was reduced, the orientation of the nanocellulose inside one gel tended to be reduced, as pointed out in a previous report [5].

### 3.3. Patterning Results

To evaluate the orientational effect of liquid-phase 3D patterning, we programmed a unidirectional pattern with a programmed area of 18 mm × 50 mm for TNW and TNF suspensions with different concentrations, and produced films with various patterning conditions including the needle diameter and discharging and patterning speeds, as summarized in Table 1 and Figure S1. The unidirectionally patterned films made of TNWs exhibited larger orientational order parameters than the films made of TNFs. This tendency is thought to be because of the nanofiber morphology, where rod-like TNWs more easily aligned than the curved TNFs (Figure 1c,d), as expected.

Within the TNWs, a slight dependence of the suspension concentration was observed: a higher concentration resulted in a higher orientation. Furthermore, from the current results, we inferred that the thickness of the dried film largely affects the final orientation. The thicker films tended to show larger order parameters. The film thickness was found to be strongly affected by the gel shape just before drying. One possible reason is that the patterned gel filaments did not strongly adhere to each other, although each filament was gelated, and the center part of the unidirectionally patterned gel sometimes deformed as the internal acetone flowed out by gently removing from the acetone bath. The W6 sample was programmed to laminate 50 layers of gel filaments, and during removal from acetone, the filament alignment spread out to deform the gel shape (see Figure S1f). However, at least during patterning, the gel shape did not collapse. The W5 film with 40 layers of gel filaments without collapse was 64 µm thick, whereas the W6 film with 50 layers was only 59 µm thick due to collapse.

In addition, the 2D film formation did not allow for the gel width to shrink upon drying to enhance their orientations unlike the one-dimensional (1D) spun fibers [4,5,6,7] or thin walls of 3D cellular architectures [21]. Although there is still technical room for further enhancing the orientation, the liquid-phase 3D patterning technique was demonstrated to produce nanocellulose orientation as programmed patterns.

### 3.4. Multiaxis Oriented Nanocellulose Film

**Figure 3:**
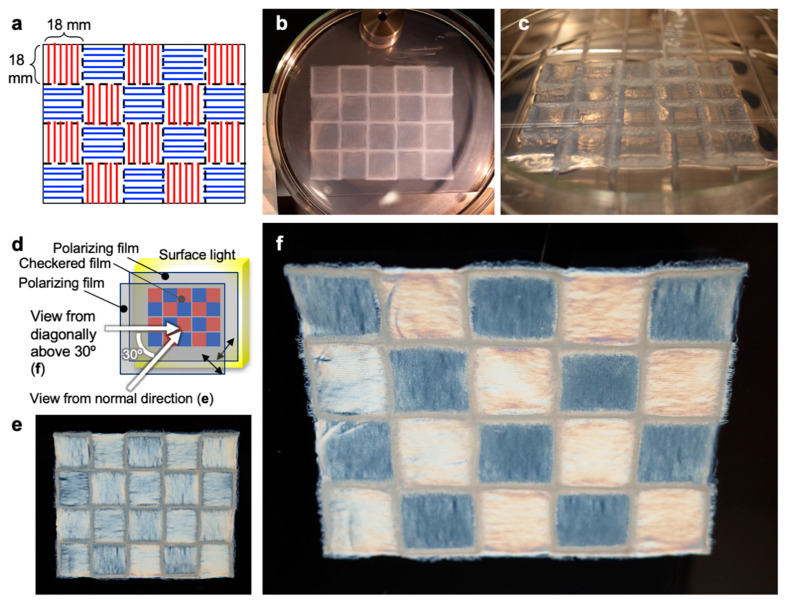
Checkered film of multiaxis oriented nanocellulose. (**a**) Programmed design of the checkered multiaxis oriented nanocellulose film. 10-layer patterned TNW gels in (**b**) an acetone bath from a 1.16 wt% suspension using a 25G needle and discharging and patterning speeds of 3.9 µL/s and 90 mm/s, respectively, and (**c**) dried in a 50 °C oven after removing extra acetone. (**d**) Birefringence observation scheme. Checkered film viewed in the (**e**) normal direction and (**f**) from diagonally above 30° from the normal direction under crossed Nicols.

To highlight the features of 3D patterning, we produced multiaxis oriented films by programming a checkered pattern combining orthogonal patterning directions in each pattern (Figure 3a). The TNW suspension was successfully patterned as programmed (Figure 3b) and then dried in an oven after the extra acetone was gently removed to obtain a freestanding checkered film. The film was sandwiched between two polarizing films with orthogonal polarizing axes (Figure 3d). When the film was observed from the normal direction, strong birefringence was equally observed in every domain. This is because the checkered domains had the same 45° angle relative to both polarizing axes. Conversely, by moving the viewpoint diagonally above at 30°, a clear birefringent checkered flag pattern appeared, as shown in Figure 3f. Regardless of the relatively small orientational order parameters (Table 1), the checkered film was demonstrated to show clear multidirectional birefringence. Similar multidirectional birefringence was also observed for the checkered film of multiaxis oriented TNF films.

## 4. Discussion

**Figure 4:**
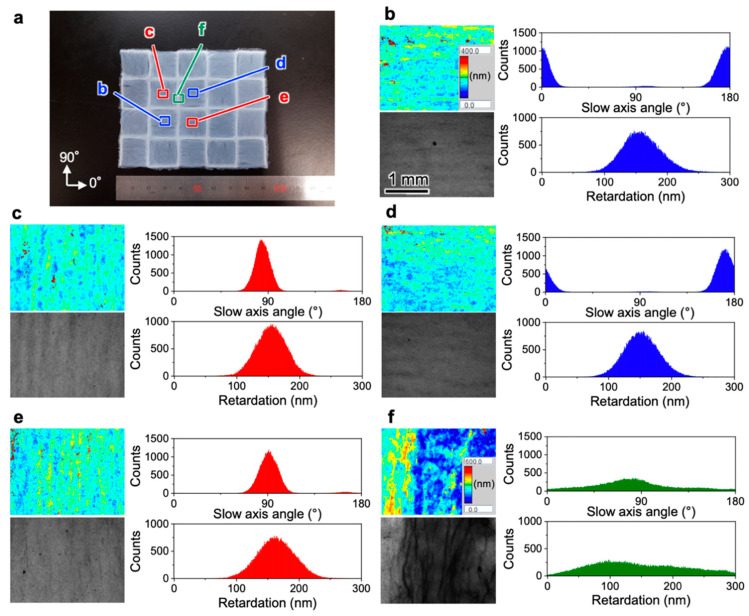
Local retardation of the checkered film of multiaxis oriented TNWs. (**a**) Location and angle definition for the retardation measurements in (**b**–**f**); (**b**–**f**) 2D retardation maps (upper left panel), brightness images (lower left panel), slow axis angle distributions (upper right panel), and retardation distributions (lower right panel). The color scales of the retardation maps for (**b**–**e**,**f**) correspond to the inserts in the upper left panels of (**b**,**f**), respectively. All of the scales of the retardation mappings and brightness images correspond to the scale of the lower left panel in (**b**).

The birefringence of the checkered TNW film was quantitatively evaluated. The retardation distribution of each domain in the checkered film was separately measured (Figure 4a). The distributions of the slow axis, corresponding to the cellulose molecular chain direction, were consistent with the patterned directions. The domains in Figure 4b,d with the 0° patterning direction showed average slow axis angles of 177.2 ± 14.3° and 172.1 ± 11.4°, whereas the domains in Figure 4c,e with the 90° patterning direction showed average slow axis angles of 84.7 ± 10.4° and 91.4 ± 14.1°, respectively. In addition, all of the domains had similar retardation values: 163.5 ± 42.3 nm, 156.5 ± 31.0 nm, 154.3 ± 33.7 nm, and 167.3 ± 41.1 nm for the areas in Figure 4b–e, respectively. Only the boarder part between domains showed broad distributions of both the slow axis and retardation (Figure 4f). The liquid-phase 3D patterning technique were demonstrated to have high repeatability to form the multiaxis oriented films with uniform optical retardation and the different birefringence axes as programmed.

**Figure 5:**
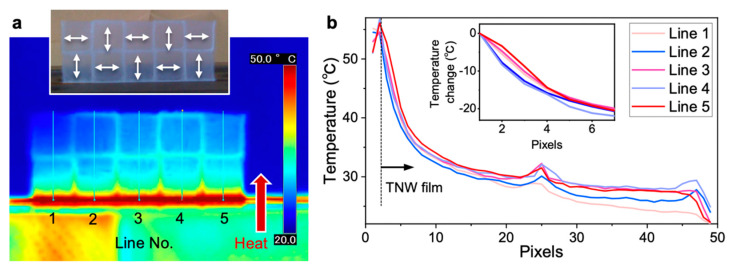
Thermal transport properties of the checkered film of TNWs. (**a**) Thermograph of the film cantilevered by a hot-press machine at 60 °C. The insert shows a photograph of the cantilevered film with the patterning directions indicated by double-headed arrows. (**b**) Temperature profile on each line defined in (a) from the hot-press machine at the pixel of line 1. The insert shows the relative temperature change in each line from the holding end of the film.

The thermal transport performance of the checkered TNW film was also evaluated. The previous studies demonstrated that the thermal conductive properties of nanocellulose films depend on the crystalline width of cellulose I [24], bulk density [28], and nanocellulose orientation [10]. Half of the checkered film was cantilevered in a hot-press machine at 60 °C and the temperature distribution was observed (Figure 5a). The tiled domains were clearly observed, and the heat transferred farther in the domains with the pattern parallel to the direction in which heat was transmitted.

To specifically visualize the difference in heat transfer, we extracted the temperature data on the lines defined in Figure 5a, and the relative temperature change was calculated (insert of Figure 5b). In the domains with a parallel pattern to the heat flow (lines 1, 3, and 5 with reddish colors), heat propagated farther than in the domains with the orthogonal pattern (lines 2 and 4 with bluish colors). The maximum difference of the temperature change of ~5 °C was observed for the checkered TNW film. Interestingly, this difference was similar to the temperature difference of ~6 °C between a nonwoven TNW film and a polyimide film [24]. A similar result was observed for the checkered TNF film (Figure S2), although the maximum temperature difference was ~2 °C because of the smaller orientation degrees. The present results agreed well with the previous findings [10,24]. We have demonstrated that thermal transport can be managed by multiaxis oriented nanocellulose films.

## 5. Conclusions

We produced checkered films with multiaxis oriented nanocelluloses by the liquid-phase 3D patterning technique combining wet spinning and 3D printing. The checkered films showed different birefringence axes with similar retardations, as programmed. The thermal transport was enhanced in the domain with the parallel direction to the heat. In addition, the patterning technique also realized programmed lamination of multiaxis oriented nanocelluloses (Figure S3). This lamination is not like chiral nematic self-assembly and has a separately designed orientation axis in each layer, which allows for the development of circularly polarizing films. This liquid-phase 3D patterning technique could pave the way for the bottom-up design of differently aligned nanocellulose films to develop sophisticated optical and thermal materials.

## Figures and Tables

**Table 1:** Results of liquid-phase 3D patterning of nanocelluloses.

Type of Nanocellulose and Name		Concentration of the Discharging Suspension (wt%)	Syringe Needle Diameter (mm)	Discharging Speed of the Suspension (µL/s)	Patterning Speed of the Three-Axis Robot (mm/s)	Oven Drying Temperature (°C)	Thickness of the Dried Film (µm)	Orientational Order Parameter *S*
TNF		~0.5	0.25	2.4	50	50	52	0.07
				3	71		38	0.08
				3.6	82		45	0.07
				3.9	90		36	0.07
						80	53	0.09
TNW	W1	0.40	0.12	1	90	50	21	0.10
	W2	1.22		1			136	0.16
	W3			1			53	0.10
	W4			1			51	0.09
	W5		0.17	2			64	0.11
	W6		0.25	3.9			59	0.14
	W7	0.91	0.12	1			52	0.13

## Supplementary Materials

The following are available online at https://www.mdpi.com/2079-4991/10/5/958/s1, Figure S1: Production of unidirectionally oriented TNW films with programmed area of 18 mm × 50 mm: (**a**) W1, (**b**) W2, (**c**) W3, (**d**) W4, (**e**) W5, (**f**) W6, and (**g**) W7. Each figure includes the appearance of the patterned gel (upper panel) and birefringence image under crossed Nicols (lower panel) along with an insert showing the 2D-XRD reflection image, Figure S2: Thermal transport properties of the checkered film of TNFs. (**a**) Thermograph of the film cantilevered by a hot-press machine at 110 °C. The insert shows a photograph of the cantilevered film with the patterning directions indicated by double-headed arrows. (**b**) Temperature profile on each line defined in (**a**) from the hot-press machine at the pixel of line 1. The insert shows the relative temperature change in each line from the holding end of the film, Figure S3: Programmed lamination of multiaxis oriented nanocellulose patterning, Video S1: Unidirectional patterning of the nanocellulose suspension.
