# Supplementary material for: Checkered Films of Multiaxis Oriented Nanocelluloses by Liquid-Phase Three-Dimensional Patterning
Source: Nanomaterials (Basel). 2020 May 18;10(5):958. doi: 10.3390/nano10050958 (PMC7281742; doi:10.3390/nano10050958)
Supplement: Supplementary file 1 [file nanomaterials-10-00958-s001.zip › SI/20200408_SI_02.pdf]

Supporting Information

Checkered films of multiaxis oriented nanocelluloses by liquid-phase three-dimensional patterning

Kojiro Uetani [1,*], Hirotaka Koga [1] and Masaya Nogi [1]

[1] The Institute of Scientific and Industrial Research, Osaka University, Mihogaoka 8-1, Ibaraki-shi, Osaka 567-0047, Japan; uetani@eco.sanken.osaka-u.ac.jp (K.U.); hkoga@eco.sanken.osaka-u.ac.jp (H.K.); nogi@eco.sanken.osaka-u.ac.jp (M.N.)

* Correspondence: uetani@eco.sanken.osaka-u.ac.jp; Tel.: +81-6-6879-8442 (K.U.)

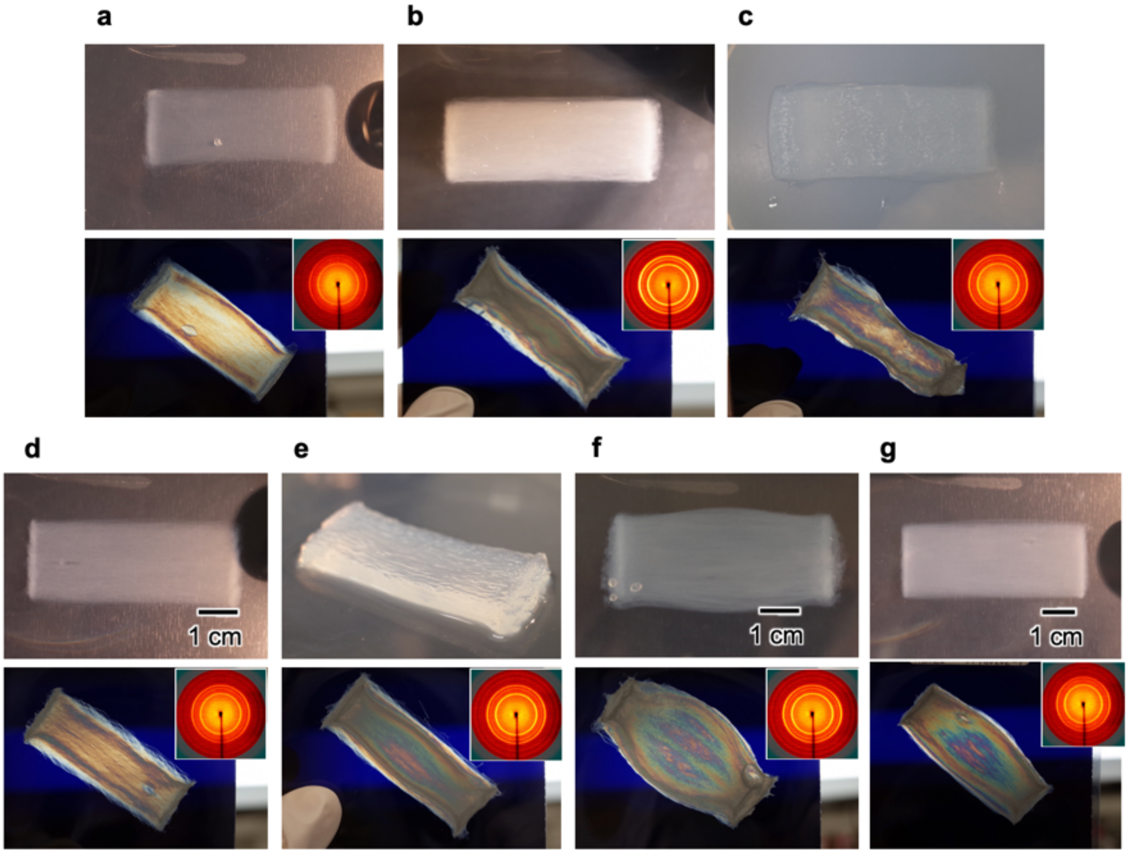

Figure S1. Production of unidirectionally oriented TNW films with programmed area of 18 mm × 50 mm: (**a**) W1, (**b**) W2, (**c**) W3, (**d**) W4, (**e**) W5, (**f**) W6, and (**g**) W7. Each figure includes the appearance of the patterned gel (upper panel) and birefringence image under crossed Nicols (lower panel) along with an insert showing the 2D-XRD reflection image.

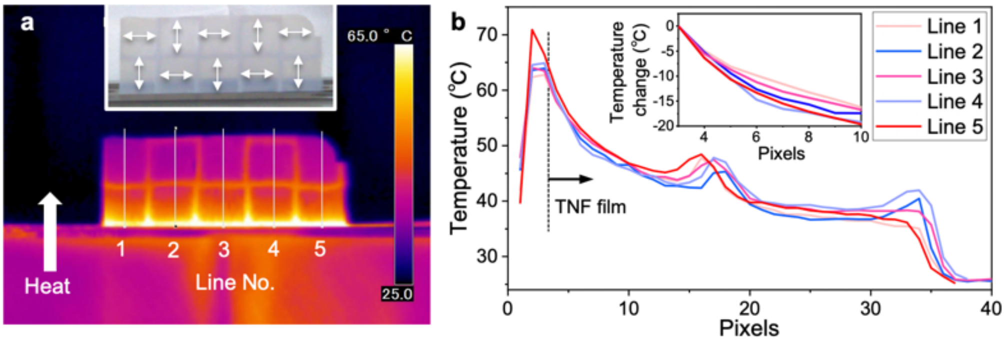

Figure S2. Thermal transport properties of the checkered film of TNFs. (**a**) Thermograph of the film cantilevered by a hot-press machine at 110 °C. The insert shows a photograph of the cantilevered film with the patterning directions indicated by double-headed arrows. (**b**) Temperature profile on each line defined in (a) from the hot-press machine at the pixel of line 1. The insert shows the relative temperature change in each line from the holding end of the film.

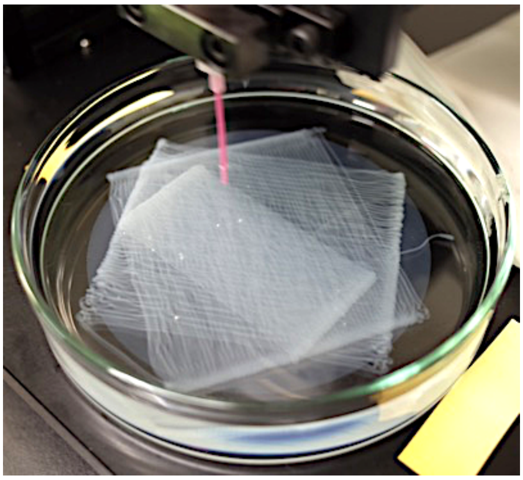

Figure S3. Programmed lamination of multiaxis oriented nanocellulose patterning.
